# Quality of Life and Community Wellbeing of Members Associated With Village Savings and Loans Associations as a Model of Sharing Economy in the Least Developing Countries: A Case of Mzuzu City in Northern Malawi, Southern Africa

**DOI:** 10.3389/fpsyg.2022.764959

**Published:** 2022-02-28

**Authors:** Xue-Lian Wu, George N. Chidimbah Munthali, Mastano N. Woleson Dzimbiri, Abdur Rahman Aakash, Muhammad Rizwan, Yu Shi, Gama Rivas Daru, Wegayehu Enbeyle Sheferaw

**Affiliations:** ^1^School of Economics and Management, Yangtze University, Jingzhou, China; ^2^Finance Department, Mzuzu University, Mzuzu, Malawi; ^3^College of Education, Health, and Society, Miami University, Oxford, OH, United States; ^4^Statistics Discipline, Science, Engineering and Technology (SET) School, Khulna University, Khulna, Bangladesh; ^5^Department of Statistics, Mizan-Tepi University, Tepi, Ethiopia

**Keywords:** quality of life (QOL), village savings and loans associations (VSLAs), living standards, rural economic growth, wellbeing and happiness, sub-Saharan African countries

## Abstract

This study was aimed at examining the impacts of the Sharing economy on the individual and community Quality of Life (QOL) and wellbeing by looking at their associated influencing factors using Village Savings and Loans Associations as a model of sharing economy in Malawi. An online community-based cross-sectional study design was conducted from November 2020 through January 2021. In the survey, 402 Village Savings and Loans Associations (VSLAs) members from the Mzuzu City area participated, recruited using snowball and respondent-driven sampling techniques. The sample size was computed using a single population proportion using the Yamane formula. Descriptive statistics and ordinal logistic regression model techniques were also employed. Additionally, we used the Chi-Square test, two-way ANOVA, and Ordinal regression model to determine statistical associations between socioeconomic data and QOL and wellbeing variables with a 5% level of significance. On the aspect of community wellbeing, the findings of our study indicated that income (levels and disposal) provided members with options to live a better QOL and wellbeing within the community by either facilitating payment for better education, eating healthier foods, acquiring assets, etc. Further, the absence of discrimination provided a platform for voice, inclusion, and social trust, enhancing freedom of expression. We also found that education facilitated better earnings and knowledge of public health-related issues. As for the contribution to the sharing economy, our study has emphasized the role played by trust in enhancing sharing economy. We recommend and encourage people to join these VSLAs so as to improve their QOL and wellbeing. However, there is a need to replicate the study on a larger scale to validate our study findings for effective policy formulation and implementation geared to improving the overall quality of people’s lives. Based on these findings, we further recommend that authorities reinstate programs like National Strategy for Financial Inclusion 2016–2020 and Savings and Loan Groups Best Practice Guidelines (SLG BPGs) 2016–2017 that could further enhance the future of VSLAs, which are vital for QOL and community wellbeing of the people in developing countries like Malawi.

## Introduction

Current statistics indicate that the value of sharing economy has grown and will continue to grow for the upcoming years (Yi et al., 2020; Barile et al., 2021). For instance, it is projected that its value will grow from 2014’s 15 billion U.S. dollars to 335 billion U.S. dollars by 2025 (Statista, 2020). The sharing economy has been associated with concepts including the gig economy, the on-demand economy, and access-based consumption (Köbis et al., 2021). Whereas, by definition sharing economy is *“a socio-economic system enabling an intermediated set of exchanges of goods and services between individuals and organizations which aim to increase efficiency and optimization of sub-utilized resources in society.*”(Muñoz and Cohen, 2017, p. 1). Further, other scholars like Stephany as cited by (Agarwal and Steinmetz, 2019, p. 3) defined sharing economy as “*The sharing economy is the value in taking underutilized assets and making them accessible online to a community, leading to a reduced need for ownership of those assets.*”

Having such global social-economic impacts, this new concept of sharing economy has attracted the attention of many scholarly contributions and industry professionals from all fields of learning and business. Recently, studies have been conducted in the fields of tourism where they tried to explore the experience of sharing economy in accommodation (Zhang and Fu, 2020), and others tried to explore the impacts of sharing economy on urban sustainability (Enochsson et al., 2021). In addition, other studies have explored the developments of the local public sector sharing economy (Cheetham and Lever, 2021), whereas, others have further explored the use of sharing economy on social inclusion and wellbeing (Davlembayeva et al., 2020), and whether Sharing Economy Platforms (SEPs) enhances the relation of sustainable innovations in the business ecosystems (Rong et al., 2021), etc. However, as there is already such a volume of literature extant, there is still a need for studies on the impacts of sharing economy on individual and community wellbeing.

Currently, there is limited knowledge on the impact of sharing economy on individual or community wellbeing especially using the Village Savings and Loans Associations (VSLAs) models in many least developing countries like Malawi. To fill this research gap, our study investigates the impacts of the Sharing economy on the individual and community Quality of Life (QOL) and wellbeing by looking at the associated influencing factors using VSLAs as the model of sharing economy. We achieved this goal by addressing the following research questions: (1) What is the current socio-economic status of VSLAs members concerning QOL and wellbeing, (2) What factors influence the QOL and wellbeing of VSLAs members. Our first null hypothesis is that most of the members do not live under better QOL and wellbeing while the alternative hypothesis is that the majority of the members have a better QOL and wellbeing. The second null hypothesis is that there is no relationship between the social-economic factors and QOL and wellbeing of VSLAs members while the alternative hypothesis is that the social-economic factors influence the QOL and wellbeing of the VSLAs members.

We have structured and organized the least of this article as follows; in continuation with the first section, we have provided an extensive literature review on the; (i) evolution of sharing economy, (ii) the evolution of VSLAs and their relationship to sharing economy, and lastly (iii) then the concepts of QOL and community wellbeing. In the second section, we have presented the methodology of data collection, In the third section, we have presented the results of the study using tables and charts, then in the fourth section we have given our discussion, and in the last section, we have provided a conclusion and recommendations to the policymakers.

### Literature Review

#### The Evolution, Aspects of Sharing Economy, and Its Connections to Village Savings and Loans Associations

There is vivid evidence in the literature that sharing economy has and is still witnessing rapid growth around the world. Despite the recent growth of this concept, there is no clear indication from present literature as to when exactly this concept was first conceived. Some scholars attribute its conception to the birth of eBay in the year 1995 when it was launched online (Belyh, 2019). First referred to as a peer-to-peer (P2P) sharing service, the concept has taken a broader definition nowadays. This has also brought confusion as many terms have been associated with this concept across the world. However, other scholars have pointed out that caution must be taken to differentiate sharing economy and related concepts like P2P, as the sharing economy is said to help to enhance the other concepts (Pasimeni, 2021).

By the analysis of the literature, it is revealed that sharing economy comes in many aspects, some of them being; (i) collaboration online, (ii) social commerce, (iii) online sharing, (iv) ideological considerations (Hamari et al., 2016). Furthermore, other scholars have identified seven dimensions of business models that fall under sharing economy. These dimensions are platforms for collaboration, under-utilized resources, peer-to-peer interactions (P2P), collaborative governance, mission-driven, Technology reliance (Muñoz and Cohen, 2017). Whereas, on its aspects, literature shows that the concept of sharing economy is based on these elements, (i) social-system, (ii) exchange of under-utilized goods and services (trading) aimed at efficient usage of the resources especially underutilized ones, and lastly, (iii) the involvement of online platform or means and usage on the internet to facilitate transactions. These three key elements are very vital in the establishment and the running of each and every sharing economy model. It is therefore not surprising to note that one of the factors that have enhanced the boom of this model is the advancement in technological development through the internet and other online communication channels, with increasing emphasis on the use of big data (Hossain, 2020; Pasimeni, 2021). Literature indicates that this advancement in technology has helped players on the market, be either suppliers or service providers in the sharing economy it has facilitated them to provide their goods and service easily and promptly. On the consumers’ end, technical advances have improved the access and enjoyment of relevant goods and services, and there is more convenient consumption at more affordable prices (Pasimeni, 2020, 2021).

On the note of enhancement factors, there is much evidence in the literature that the financial and economic crisis of late 2008 had also positively contributed to the boom of this model (McCrea et al., 2019; Hossain, 2020). This was so, because it’s said that during the crisis the market players wanted to be secured in trade by either making a profit on underutilized resources for suppliers or avoiding the risk inherent in having continual ownership of goods and services for consumers (Habibi et al., 2017). Aside from these factors that have contributed to the boom, there are still more reasons why many stakeholders have joined and are supporting this model. Being within a competitive business industry, the support has even helped them to be sustained. Projections for future growth are also positive (Statista, 2020). Some of these reasons are economic gains from stakeholders, environmental conservation from policymakers and environmentalists, sustainability which is linked directly to the positive attitude of the group members, and social capital (Hamari et al., 2016; Mi and Coffman, 2019; Hossain, 2020). Though numerically many people have indicated that have joined and participated in these models, some scholars have cautioned that there are still some negative impacts or consequences that have arisen from this business model as there have been some traces of negligence that arise through poor regulations and policies that are governing these models in certain economies (Hossain, 2020; Köbis et al., 2021).

Based on this theoretical background of the sharing economy, especially in regard to peer-to-peer (PP) interaction, it satisfies the relevance and influence that sharing economy has on savings groupings like of VSLAs, which can be argued, and seen to be categorized under the sharing economy models. It is therefore clear and evidenced that these VSLAs fall under the channels or models of sharing economy in one way or another, for example, by looking at their aims of formation, their setups, etc. Just like sharing economy models, in VSLAs trust is one of the pillars on which they are built, based, and rooted (Maganga, 2020; Köbis et al., 2021). Meaning that a lack of trust possess a threat to both sustainability and implementation (Govindan et al., 2020), which is exactly the same in the sharing economy where you’re not able to physically see the owners or providers of the goods or services you enjoy but based on trust and other factors your ability to transact with one another. Apart from trust, the sharing economy and VSLAs both are influenced by social capital elements due to their interlinks to e-commerce and usage of the internet within a community or society (Lin et al., 2020; Wei et al., 2020).

#### Developments of Village Savings and Loans Associations Models and Their Impacts on Quality of Life and Community Wellbeing

Developed and developing countries differ in their approaches toward improving the QOL and wellbeing of their citizens. In many developing countries in Sub-Saharan African and other parts of Asia, like Malawi, Zambia, Uganda, Ghana, Vietnam, and Nepal, etc. the physical approaches such as savings groups are the most common models that are used as tools for eradicating extreme poverty, fighting chronic hunger, social and economically empowering those groups of people who are traditionally marginalized (Maganga, 2020; Otekunrin et al., 2020a,b). There are many models of saving groups formed around the world with the common and highly popularly one being Village Savings and Loans Associations (VSLAs), which is said to be first introduced in 1991 by the Cooperative for American Remittances to Europe (CARE) in Niger (Galvao et al., 2012; Karlan et al., 2017, 2018). Since then, CARE VSLAs model groups have been introduced in many developing economies, especially in Africa and some parts of Asia. Malawi has been no exception. Despite having VSLAs models by CARE, these savings groups are largely run as informal settings; they are known and named under different programs as preferred by their environment or community for ease of communications across the countries.

For instance, in Malawi, these groups are named *Mudzi Bank* or “*Bank M*’*mudzi*,” meaning village banks (MBC, 2021), whereas, in Egypt, they are called Rotating Savings and Credit Associations (RSCA) (Shaaban, 2019), Group Savings and Loan Associations (GSLAs) in Kenya (Matthews and Green, 2010), and Livelihood Enhancement and Associations among the Poor (LEAP) in Cambodia (Ban et al., 2020). In this study, we have adopted the concept of VSLAs to mean savings groups, including all the savings groups known to be operating in Malawi. According to CARE, a Village Savings and Loan Association (VSLA) is a group of 10–25 people who come and save money together on either a weekly or monthly basis, and members are allowed to acquire small loans commensurate with the money saved in their accounts (Ngegba et al., 2016; Hinson et al., 2017). The activities of the VSLAs take about 1 year, after which the accrued savings and profits are shared out among the members based on how much money they have saved (Staehle, 2011).

The concepts of VSLAs, QOL, and wellbeing have been widely applied together in different setups. In many VSLA studies, the majority of the scholars have explored areas surrounding the impact of VSLAs on the social-economic development especially in developing countries like those found in the Sub–Saharan Africa (SSA) region, which measures the subjective wellbeing of the individual and community at large (Hendricks and Chidiac, 2011; Bruton et al., 2015; Jayaram and de, 2021). For example, studies have demonstrated that VSLAs have an impact on eradicating malnutrition in children under the age of five, thus enhancing the health wellbeing of the children within a community (Brunie et al., 2014). A study conducted in Ghana on the adoption of agricultural technologies by farmers revealed that VSLAs had a positive impact on the adoption rate of these agricultural technologies by helping in capacity building for farmers, as many of them farmers were able to save and buy the farming inputs with the help of the saving loans or profits gained (Dagunga et al., 2020), which in turn also helped them to optimize their agriculture production such that food sufficiency was realized. Regarding our current focus, QOL and community wellbeing status, the majority of studies have been done in the health discipline (occupation health and epidemiology setups), whereby examining the impact of different underlying health conditions on QOL among patients (Soriano et al., 2016; Mesafint et al., 2020), and also the QOL among aged individuals (Escuder-Mollon, 2012). In addition to that, other studies have been conducted to examine the QOL among the migrant population (Bobek, 2020), the mobility of people in general (Kolodinsky et al., 2013), the medical practitioners like nurses (Nursalam et al., 2018) and also among students and adolescents in schools (Geng et al., 2020), with a limited number of studies being conducted to examine the QOL and wellbeing in workplaces and business setups (Kolodinsky et al., 2013). Thus, there is a gap in knowledge as the majority of these studies have been conducted on an individual basis, with the focuses being certain health conditions like chronic diseases or social groups like women and the elderly. We find it crucial to also find out the impacts of these sharing economy models of VSLAs on QOL and the wellbeing of members at the community level. Integrating these concepts and finding how they are mutually inclusive is critical to addressing not only academics, but also policymakers, NGOs as well as the private sector forces working in VSLAs that want to strengthen their operations in regards to poverty eradication, nutrition, and economic empowerment, etc.

#### The Concepts of Quality of Life and Community Wellbeing

Quality of Life (QOL) and wellbeing are some of the most popular and broad topics or concepts in the disciplines of the social sciences that have been studied for decades at both micro and macro levels in various disciplines (McCall, 1975). The main reason these topics have received such considerable interest from many scholars is their focus on human life and development in general. For instance, in the case of QOL, a person who enjoys a better QOL is said to be productive, and such an individual is quite likely to positively contribute to the country’s social-economic development (The WHOQOL Group, 1998). Furthermore, on the aspect of wellbeing, an individual with higher wellbeing is also said to be more productive and creative, thus, contributing to the development of the community and the country’s economy in general (Ruggeri et al., 2020). The concept of QOL has been closely associated with human happiness and wellbeing. However, some scholars argue that it evidently transcends the two aforementioned concepts. QOL is defined variously across disciplines. In other words, it has a legion of different meanings depending on the discipline in which it is being examined. In health sciences, the World Health Organization (WHO) defines QOL “*as an individual*’*s perceptions of their position in life in the context of the culture and value systems in which they live and in relation or concerning to their goals, expectations, standards, and concerns*” (The WHOQOL Group, 1998, p. 5551). While other scholars like Rice (1984, p. 157) as cited by (Livingston and Fink, 2003) define QOL “*as the degree to which the experience of an individual’s life satisfies that individual’s wants and needs (both physical and psychological)*” (Livingston and Fink, 2003, p. 383). On the other hand, many scholars also differently define wellbeing. For example, Kai defined wellbeing “*as the combination of feeling good and functioning well; the experience of positive emotions such as happiness and contentment as well as the development of one*’*s potential, having some control over one*’*s life, having a sense of purpose, and experiencing positive relationships*” (Ruggeri et al., 2020, p. 1).

There is much evidence that QOL and Community wellbeing concepts have been used interchangeably by many scholars. It is evidenced that these two concepts can be measured either subjectively, including self-reported levels of life satisfaction, happiness, etc., or objectively, which includes literacy rates, life expectancy, etc., respectively (Costanza et al., 2007; Bec et al., 2016; McCrea et al., 2019). There has been debate in the academic circles which has left no clear concession as other scholars argue that their line of differentiation is more about illusory, as they see QOL as a subjective measure of wellbeing (Costanza et al., 2007). Whereas, the measurements of objective QOL are characterized by social, economic, and health indicators. Based on the definition, Forzaj defined community wellbeing as *“the satisfaction with the local place of residence taking into account the attachment to it, the social and physical environment, and the services and facilities”* as cited in (McCrea et al., 2019, p. 88). Their study also pointed out that community wellbeing is more a perceived QOL, which is demonstrated and measured at a community level. Babra further defined QOL *as* “*the general wellbeing of individuals and society whereby wellbeing is measured into two dimensions of subjective and objective*” (Tobiasz-adamczyk, 2014, p. 3–4). Thus, there are strong links of evidence in the concepts of QOL and Community wellbeing especially based on the attributes of measurements they share (Pinto et al., 2017).

As discussed, associations enhance community wellbeing (Gillam and Charles, 2019). Therefore, this may mean that associations like VSLAs have the capability of impacting community wellbeing. In this study, we used the term QOL to encompass the community wellbeing concept and integrated the subjective and objective measurements to get a deeper understanding of the impact of the VSLAs on its members. To achieve our goal, we have defined QOL as “*the extent to which objective human needs are fulfilled in relation to personal or group perceptions of subjective wellbeing*” (Costanza et al., 2007, p. 268), with all cautions being in place to mark the differentiating lines of the two concepts which are advised to be taken care of (Salvador-Carulla et al., 2014; Pinto et al., 2017).

## Materials and Methods

### Study Area

**FIGURE 1:**
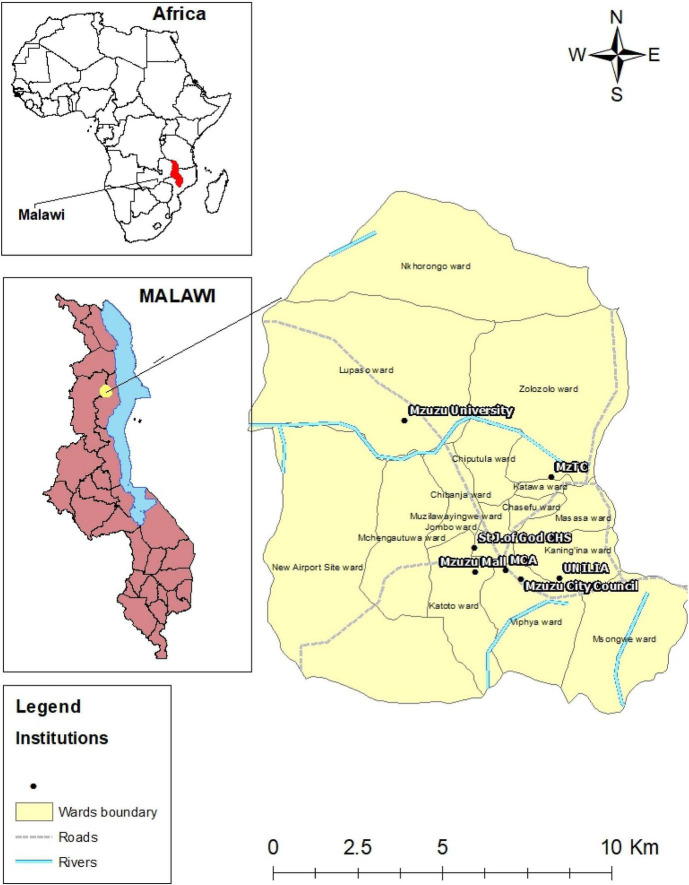
Map showing study localities within Mzuzu city, Malawi. Source: Created by authors in ArcGIS version 10.61.

This study was conducted in Mzuzu city, situated in the northern part of Malawi (Figure 1). Malawi is situated in the South-East of Africa, sharing borders with Zambia to the west, Tanzania to the north, and Mozambique to the south. The country has an estimated total population of about 17,563,749 (National Statistical Office [NSO], 2019; Munthali and Xuelian, 2020; WPR, 2020). Mzuzu city has a total area of 143.8 square kilometers and is divided into 14 political boundaries known as wards (Table 1) with an approximate total population of about 221,272 (National Statistical Office [NSO], 2019). The city is ranked as the third-largest administrative city in Malawi, which is currently experiencing rapid urbanization. According to the current International Food Policy Research Institute (IFPRI) report, Mzuzu City is ranked and categorized in the group of the districts that have indicated a low incident of poverty of about 9.7%, second from Blantyre City (IFPRI, 2019). The population of Mzuzu is mixed with all classes of people and characterized by various socioeconomic activities which have contributed to this rapid growth. Some of the socioeconomic activities that have contributed to this urban growth include numerous agricultural activities such as tobacco farming, fish farming, and the growing of coffee by the Mzuzu Coffee Company. Mzuzu city is also well known as an academic hub that is home to various institutions of higher learning, which contribute enormously to the social and economic development of the country as a whole. These institutions include colleges and universities like Mzuzu University (MZUNI), University of Livingstonia (UNILIA), St John of God College of Health Sciences, Malawi College of Accountancy (MCA), and Mzuzu Technical College (MzTec) among others, due to these factors Mzuzu city has recently registered a boom in much emerging small scale business operations (UN-HABITAT, 2011; National Statistical Office [NSO], 2019). Therefore, the city was selected for our study because of its remarkable impacts on the country’s development in terms of economic growth and development as well as the presence of numerous VSLAs or savings groups that are in operation across the Mzuzu city.

**TABLE 1:** Wards distribution of data (*N* = 402).

Location	*f*	%
Chibanja	12	3.0
Chibavi East	20	5.0
Chibavi West	13	3.2
Chiputula	15	3.7
Jombo-Kaning’ina	15	3.7
Katawa	12	3.0
Lupaso—Nkhorongo	6	1.5
Luwinga	48	11.9
Masasa	96	23.9
Mchengautuwa East	57	14.2
Mchengautuwa West	64	15.9
Mzilawaingwe	17	4.2
Zolozolo East	20	5.0
Zolozolo West	7	1.7

*Source: Field survey, 2021.*

### Measure of Variables

We used QOL and wellbeing as the dependent variable. QOL and wellbeing were measured by the satisfaction of life by the members of the VSLAs in Mzuzu. The satisfaction was measured by using a scale ranging from (1–3) Least/Low satisfied, Moderate or Medium, and Highly satisfied (see Table 2). The independent variables included demographic, economic data, and the control variables were disposable income, education fund, discrimination, and freedom of expression. The demographic and economic data included gender, age, occupation, education, house roof, assets, location, registration, bank account, and income. The measures of all the social and other variables are defined in Table 2.

**TABLE 2:** Description, definition, and values of variables used in the ordinal regression model.

Research variables	Definitions	Value and unit of measurements	Citations
Gender	Gender of the members	Dummy variable, 1 = male, 0 = female	Atube et al., 2021
Age group	Age group of members	Categorical, years 1 = <30, 2 = ≥31 years old	
Occupation	Primary occupation of members	Dummy, 1 = Employed, 0 = Otherwise	
Education	Education level of the members	Dummy, 1 = Attended, 0 = Otherwise	
House1	Are you head of the house	Dummy variable, 1 if yes, 0 otherwise	
House number	Number of people in the house	Categorical, 1 = <5, 2 = ≥6	
House status	Do you own or rent the house	Dummy variables, 1 if yes, 0 otherwise	
Roof	What is the roof of your house made up of	Categorical, 1 = iron sheet, 0 = Otherwise	
Own asset	Do you own these assets furniture, wires, tv	Dummy variable, 1 if yes, 0 otherwise	
Registration	Is your group registered	Dummy variable, 1 if yes, 0 otherwise	
Bank account	Do you have a bank account	Dummy Variable, 1 if yes, 0 otherwise	
Income	Average monthly income earnings of members	Categorical, 1 = < MK5,000, 2 = < MK5,000	
Quality of life and wellbeing (DV)	What was the respondent ranking of their quality of life and wellbeing due to the coming of VSLAs in Mzuzu city	Categorical, 1 = Poorly/Low satisfied, 2 = Moderate satisfied/Impacted, 3 = Highly Satisfied/Impacted	
Disposal income	VSLAs led to increase disposal income	Categorical, 1 = SD, 2 = D,3 = N,4 = A, 5 = SA	Campbell, 1976
Education 2	VSLAs led to readily accessible funds for Edu.	Categorical, 1 = SD, 2 = D,3 = N,4 = A, 5 = SA	
Discrimination	VSLAs led to reduce discrimination in any	Categorical, 1 = SD, 2 = D,3 = N,4 = A, 5 = SA	
Freedom of expression	VSLAs led to improve freedom of expression	Categorical, 1 = SD, 2 = D,3 = N,4 = A, 5 = SA	

*Source: Authors, 2021.*

*MK, Malawian Kwacha ($ 1 = 780 MK); SD, Strongly Disagree; D, Disagree; N, Neutral; A, Agree; SA, Strongly Agree; VSLAs, Village Banks Savings and Loans Association; DV, Dependent Variable. MEC, Malawi Electoral Commission.*

### Study Design, Sampling, and Data Collection Procedure

We employed a quantitative approach using a cross-sectional survey design for data collection conducted between November 2020 and January 2021, a period that is popularly known to be the time that the majority of the VSLAs circles ended their sharing schemes and the new circle is started in Malawi. The inclusion criteria specified only members of the VSLAs (Village Banks = Mudzi Banks or Bank M’mudzi as popularly known by many local people) operating within 14 areas of Mzuzu City’s politically demarcated as wards. The areas in question are Chibanja, Chibavi West, Chibavi East, Chiputula, Jombo-Kaning’ina, Katawa, Lupaso-Nkhorongo, Luwinga, Masasa, Mchengautuwa East, Mchengautuwa West, Mzilawaingwe, Zolozolo East, and Zolozolo West (Table 1). Furthermore, the inclusion criteria were that the participants should; (1) agree to participate by approving on an online consent form, (2) be aged above 18 years of age (the minimum legal adult age in Malawi), and (3) have the capacity (be able) to fill and complete an online questionnaire. The exclusion criteria were self-reported locations outside of these. Such responses were not included in our analysis.

The collection of data was conducted through an online questionnaire owing to strict COVID-19 restrictions imposed by the authorities to curb the whirlwind spread of the coronavirus. Due to the informal existence of VSLAs in Malawi, the targeted population was selected by a snowball and respondent-driven sampling technique. The Snowball sampling technique is deployed in situations where there are difficulties in identifying the respondents or the subjects needed to participate in the study (Goodman, 1961; Heckathorn, 1997). In this study, we used research assistants (four members headed by Mr. Amoni Zolo a qualified researcher) who were trained in advance about the aim of the study and the way to conduct the research. These research assistants identified some of the participants associated with VSLAs in Mzuzu, Malawi. The identified participants were formally invited and all consented to take part in the study, and, subsequently, assisted in sending a questionnaire link to other VSLAs groups’ members within and outside their wards. A questionnaire link was sent *via* online platforms, namely WhatsApp, emails, and Facebook. The chosen technique was largely preferred as it was convenient, fast to reach a larger population, and cost-effective since any external sources did not fund the research.

The sampling technique is a method of taking a small ratio of observation from a large population to get information of those large populations from the sampled observation by using some statistical techniques (Bhattacherjee, 2012). The sample size (n) was calculated using the following parameters: the margin of error (e), and total population (N) will be expected. In coming up with a representative sample size, we calculated the sample using the sample size formula that was developed by Yamane (Israel, 2003), with a 95% CI and, *P* = 0.05. Taking into account the population of Mzuzu (221,272).


(1) $$x=\frac{N}{1+N\left(e\right)2}$$

$$\frac{211,272}{[(1+\left(211,272{\left(0.5\right)}^{2}\right]}$$

$$x=\frac{211,272}{544.18}$$

x=399

Where n is the sample size, *N* = (221,272) is the population size, and e = (0.05) is the level of precision. Finally, the desired sample size (339) provides the number of responses that need to be obtained for the study using Equation 1.

### Questionnaire Description

The questionnaire used for data collection had three sections. The first section captured the respondent’s demographic data such as gender, age-group, education, being a head household status, and household size. The second section captured the respondent’s economic data, such as ownership of the house, type of roof of the house, house wall, ownership of assets, registration status of the village banks, and owning a bank account. Both first and second sections of the questions collected binary responses (Yes or No), categorical variables, and ranking scale. The third part of the questionnaire consisted of variables of QOL and wellbeing domains or indicators that were adopted from the OECD handbook of QOL and wellbeing with the domain of income, education, health, discrimination, freedom of expression (OECD, 2011, 2016). Questions were asked and measured through a 5 Likert scale ranging from strongly disagree to strongly agree (Table 2).

### Statistical Analysis

Data were analyzed using IBM SPSS version 25. Descriptive data were analyzed to produce frequency tables, means, *SD*s, and charts/graphs. Pearson correlation of Chi-square and two-way ANOVA was performed to determine correlations between socioeconomic variables in which *p*-value was set at < 0.05. An Ordinal Regression Model was performed to predict factors that may influence QOL and wellbeing with a *p*-value set at < 0.05 as statistically significant.

The Ordinal Logistic Regression Model is used in cases where the dependent variable has more than two categories. Thus, in our study. We used an ordinal regression model as our dependent variable had three ordinal categories related to the satisfaction of QOL and wellbeing: Low, Moderate, and High by using Equation 2.1a–d. Test of parallel lines was used to determine whether it was reasonable to assume that the values of the location parameters are constant across categories of the response. Let Y (satisfaction of QOL) take categorical response variable with K ordered categories and assume *P*(*Y* = 1) = *P*_1_, *P*(*Y* 2) = *P*_2_,…,*P*(*Y* = *j*) = *P*_*j*_ for *j* = 1,2,3,4,5,…,*k*. Let the cumulative probability of the first of Y be *P*(*Y* ≤ *j*|*X*) = *φj*(*X*),*j* = 1, 2, 3…*K* − 1.


(2.1a) $$logit\left(P\left(Y\leq j|X\right)\right)=log\left(\frac{p\left(X\right)}{1-p\left(X\right)}\right)$$

(2.1b) $$=log\left(\frac{\varphi j\left(X\right)}{1-\varphi j\left(X\right)}\right)$$

(2.1c) $$=log\left(\frac{\varphi 1\left(X\right)+\varphi 2\left(X\right)+\ldots \ldots .+\varphi j\left(X\right)}{1-\varphi j\left(X\right)+\varphi 2\left(X\right)+\ldots \ldots .+\varphi j\left(X\right)}\right)$$

(2.1d) $$=\alpha j+X^{\prime }\beta i$$

Where, α_j_ represents the threshold (intercept), X_i_ represents the explanatory variables, and β represents the regression coefficient. Y_ik_ has three ordered categories (Low QOL and wellbeing, Moderate QOL and wellbeing, and Highly QOL and wellbeing). The ordered model estimates the cumulative probability γ_k_ or cumulative log odds $\log\left(\frac{\gamma k}{1-\gamma k}\right)$ up to the Kth category where K = 1, 2, 3. One category (last or first) of this is taken as the reference category, and cumulative probability for the reference category is always equal to one. The satisfaction rate “Low” category was the first and the satisfaction rate “High” was the last category was our research. The cumulative logit probability model takes the form $loglog\left(\frac{\gamma 1}{1-\gamma 1}\right)=\alpha 1+X^{\prime }\beta i$ and $loglog\left(\frac{\gamma 2}{1-\gamma 2}\right)=\alpha 2+X^{\prime }\beta i$.

The odds ratio for each predictor is constant across all possible collapses of the outcome variable. When a testable assumption is met, the odds ratios in a cumulative logit model are interpreted as the odds of being “lower” or “higher” on the outcome variable across the entire range of the outcome. The wide applicability and intuitive interpretation of the cumulative is the most popular model for ordinal logistic regression.

In ordinal logistic regression models, there is an important assumption that belongs to ordinal odds. According to this assumption, parameters should not change for different categories. Test of parallel lines used to determine whether it is reasonable to assume that the values of the location parameters are constant across categories of the response. If the proportional odds model is not met, there are several options which are the following: collapse of two or more levels, particularly if some of the levels have a small number of observations. Bivariate ordinal logistic analyses, to see if there is one particular independent variable that is operating differently at different levels of the dependent (McCullagh, 1980; Uyanık and Güler, 2013), partial proportional odds model, and multinomial logistic regression. For our study, test of parallel lines was satisfied.

### Validity and Reliability

Prior to the actual collection of data, the questionnaire instrument was pre-tested by conducting a pilot study that involved 43 respondents. Among these, 34 were members of the village banks in Malawi, 8 were master’s degree students, and one Ph.D. student working on economics and business studies. These were selected to participate in the pilot study because of their knowledge, experience, and expertise in the subject matter. We included nine academic respondents with the aim of critically checking technical errors, validity, and reliability of the questions being asked. We also sent the instruments to a professor of economics who also checked and screened the questionnaire. All the comments and corrections were addressed accordingly before the actual data collection exercise. The internal consistency of the instrument was validated by calculating Cronbach’s Alpha which had a result of 0.804.

## Results

Among 402 respondents who completed the survey, the majority were from Masasa ward (23.3%, *n* = 96) followed by Mchengautuwa West (15.9%, *n* = 64), Mchengautuwa East, and Luwinga (14.2%, *n* = 57) (11.9%, *n* = 48), respectively. The lowest respondents came from Lupaso-Nkhorongo and Zolozolo West (1.7%, *n* = 6) and (1.7%, *n* = 7), respectively. A large number of the wards recorded an average response rate from the participants (Table 1).

Table 3 indicates that the majority of the respondents across all the wards were male representing (64%, *n* = 259) compared to their female counterparts. Most of the respondents were within the age group of 30+ (60%, *n* = 30). In terms of education, the majority of the respondents (97%, *n* = 388) had at least attained a certain level of education such as primary, secondary, college or university whereas a few (3.5%, *n* = 14) reported having never gone to school. With regard to occupation, results demonstrated that the vast majority (71.9%, *n* = 289) were not employed and this suggests that they were in informal employment while only a few reported having been employed. Of these respondents, more than two-thirds were heads of households (*n* = 280).

**TABLE 3:** Demographic and socio-economic characteristics of the respondents (*N* = 402).

Variables	Category	*F*	%
**Gender**			
	Male	259	64.4
	Female	143	35.6
**Age group**			
	<30	157	39.1
	30+	245	60.9
**Occupation**			
	Employed	113	28.1
	Other forms	289	71.9
**Education**			
	Attended	388	96.5
	Not at all	14	3.5
**Are you head of the house**			
	Yes	280	69.7
	No	122	30.3
**Number of people in the house**			
	<5	139	34.6
	5+	263	65.4
**Do you own or rent the house**			
	Own	200	49.8
	Rent	202	50.2
**What is the rood made up of**			
	Iron sheet	376	93.5
	Not	26	6.5
**Furniture, radio, tv do you own these**			
	Yes	331	82.3
	No	71	17.7
**VSLAs registration**			
	Yes	56	13.9
	No	346	86.1
**Do you have bank account**			
	Yes	108	26.9
	No	294	73.1
**Income level**			
	<5	137	34.1
	5+	265	65.9

*Source: Field study, 2021.*

The economic status of respondents varies across Mzuzu city (Table 3). Our results show that the majority (65.4%, *n* = 263) of the respondents across all the wards earned more than MK5,000 (USD = 6.41) per month. More than half (50.2%, *n* = 202) of the respondents paid housing rentals on a monthly basis whereas nearly half reported owning the houses in which they lived (Table 3). In terms of house conditions, the majority (93.5%, *n* = 376) were living in houses made of iron sheet roofs while a few lived in grass-thatched houses. The majority (82.3%, *n* = 331) was in possession of basic household assets such as radios, television sets, and pieces of furniture like chairs and tables. Most of the VSLAs that were operational across the wards were unregistered (86.1%, *n* = 346), and most of these had no bank accounts to keep their contributions (73.1%, *n* = 294).

The bivariate analysis on demographic results depicted in Table 4 showed that variables of occupation were associated with significant differences in the scores of the QOL and wellbeing with those not working having high scores 236 as compared to those that are working (*F* = 3.867, *P* = 0.022). Whereas the bivariate analysis of social-economic indicators of Registration, with those that say Not be registered having high scores 287 (*F* = 2.551. *P* = 0.079*).

**TABLE 4:** Quality of life and wellbeing and demographic and socio-economic characteristics of Village bank members.

		QOL and wellbeing-satisfaction levels scores				χ^2^
			
Variable	Category	No/low impacted	Medium	High	*F*-value	*P*-value
Gender of the respondent	Female	24	13	**222^+^**		
	Male	21	4	118	1.805	0.166
Age group	<30	21	9	127		
	≥31 years old	24	8	**213^+^**	1.443	0.237
Education level	Otherwise	0	0	14		
	Attended education	45	17	**326^+^**	1.321	0.268
Occupation	Not employed -Otherwise	40	13	**236^+^**		
	Employed i.e., Civil servant or NGOs, etc.	5	4	104	**3.867**	0.022**
Are you head of the house	No	9	4	109		
	Yes	36	13	**231^+^**	1.563	0.211
Number of people in the house	Less than 5 members	19	5	115		
	6 members and above	26	12	**225^+^**	0.721	0.487
Do you own or rent the house	Rent	21	9	**172^+^**		
	Own	24	8	168	0.147	0.863
What is the rood made up of	Otherwise	1	2	23		
	Iron sheet	44	15	**317^+^**	1.087	0.338
Furniture, wires, tv do you own these	No	7	4	60		
	Yes	38	13	**280^+^**	0.268	0.765
Is your VB registered	No	43	16	**287^+^**		
	Yes	2	1	53	**2.551**	0.079*
Do you have a bank account	No	39	14	**241^+^**		
	Yes	6	3	99	**2.924**	0.055**
What is the average monthly income of your association during/after the outbreak of COVID-19	Less than MK5,000	2	3	132		
	Above MK5,000	43	14	**208^+^**	**12.130**	0.000***

*Source: Authors 2021 Field study.*

* Statistic significance at 0.8, ** at 0.05, and *** at 0.001.

*^+^Frequency with high values. Chi-square test.*

*Bold values indicate scores above 50% and p-values that are statistically significant.*

Secondly, those having and not having a bank account had a high score of 241 (*F* = 2.924, *P* = 0.055) and the income level of those above MK5,000 had a higher score of 208 (*F* = 12.130, *P* = 0), which found to be statistically significantly associated with the satisfaction of QOL and wellbeing at the level at which the *P*-value was set (Table 4).

To understand the effect of income variable is the strongest predictor variable as it was found statistically significant associated with QOL and wellbeing (Table 3). We further ran an extended analysis in Table 5. The results of two-way ANOVA which was used to compare the effects of income levels (less than MK5,000 and above MK5,000) on QOL and wellbeing of the Village banks members indicated statistically significant differences between all demographic variables except education and all economic characterized variables without exception (Table 5).

**TABLE 5:** Income distribution effect on QOL and wellbeing among social demographic and other related economic indicators.

		Income levels		Two way ANOVA
Variable	Category	Less than MK5,000 (frequency)	Above MK5,000 (frequency)	*P*-values
Gender of the respondent	Female	100	159	
	Male	37	**106^+^**	Income = 0.000*** Gender = 0.645 Income × Gender = 0.685
Age group	<30	41	116	
	≥31 years old	96	**149^+^**	Income = 0.000*** Age-group = 0.496 Income × Age-group = 0.814
Occupation	Not employed -Otherwise	114	**175^+^**	
	Employed i.e., Civil servant or NGOs, etc.	23	90	Income = 0.002*** Occupation = 0.044 Income × Occupation = 0.011
Education level	Otherwise	6	8	
	Attended education	131	**257^+^**	Income = 0.333 Gender = 0.202 Income × Gender = 0.333
Are you head of the house	No	49	73	
	Yes	88	**192^+^**	Income = 0.000*** House-head = 0.348 Income × House-head = 0.108
Number of people in the house	less than 5 members	42	97	
	6 members and above	95	**168^+^**	Income = 0.000*** No-of-people = 0.396 Income × Roof people = 0.596
Do you own or rent the house	Rent	54	**148^+^**	
	Own	83	117	Income = 0.000*** House-Rent/own = 0.404 Income × House-Rent/own = 0.345
What is the rood made up of	Otherwise	11	15	
	Iron sheet	126	**250^+^**	Income = 0.023** Roof = 0.506 Income × Roof = 0.812
Furniture, wires, tv do you own these	No	32	39	
	Yes	105	**226^+^**	Income = 0.000*** Assets = 0.751 Income × Assets = 0.888
Is your VB registered	No	118	**228^+^**	
	Yes	19	37	Income = 0.059** Registration = 0.139 Income × Registration = 0.033**
Do you have a bank account	No	106	**188^+^**	
	Yes	31	77	Income = 0.001*** Bank-account = 0.070* Income × Bank-account = 0.042**

*Source: Authors 2021 Field study.*

* Statistic significance at 0.8, ** at 0.05 and *** at 0.001; * Two-way ANOVA.

*^+^Frequency/score at higher level.*

*Bold values indicate scores above 50% and p-values that are statistically significant.*

Lastly, the results on ordinal linear regression on the Test of Parallel lines for the proportional odds assumption for the SPSS procedure produced an insignificant chi-square value of 4.297 with 10 degrees of freedom (*p* = 0.933). This indicates that the assumption of a parallel line was appropriate for the evidence of the data. Since we cannot reject the null hypothesis at a 5% significance level, the model satisfies the proportional odds assumption. Hence, it was not necessary to go for another model.

In this study, the idea behind fitting a proportional odds model was to assess the status and factors that influence or are associated with the QOL and wellbeing of VSLAs members. When the POM was used in the analysis of data, the co-efficient of the explanatory variables in the model were interpreted as the ratio of the odds of status QOL and wellbeing. The two intercepts were used to differentiate the category of the status of QOL and wellbeing from each comparison. −2.411 was used for comparison of moderate QOL and wellbeing to Highly QOL and wellbeing and Low QOL and wellbeing, −2.963 was used to compare category moderate QOL and wellbeing, severe QOL and wellbeing to category Low QOL and wellbeing.

Results from the ordinal regression model showed statistical significance between various socio-economic and demographic variables, demonstrating high probability in influencing the Quality of Life (QOL) and wellbeing (Table 6). Seven (7) of the significant variables included occupation (ß = −0.882, *p* = 0.045, CI = −1.745, −0.019), Income (ß = 1.291, *p* = 0, CI = 0.911, 2.932), household status (ß = 0.813, *p* = 0.045, CI = 0.02,1.607), education (ß = 19.348, *p* = 0, CI = 19.348, 19.348), disposal income (ß = 0.36, *p* = 0.002, CI = 0.129, 0.592), education funds (ß = 0.499, *p* = 0, CI = 0223, 0.775), discrimination (ß = −0.392, *p* = 0.02, CI = −0.721, −0.062) and freedom of expression (ß = −0.475, *p* = 0.037, CI = −0.92, −0.029) all variables were measured at 95% CI level. However, some demographic variables such as gender and age group of the participants were found not statistically significant as predictors of the quality of life in this model (Table 6).

**TABLE 6:** Associated factors and quality of life and wellbeing of respondents using ordinal regression model.

Variables	Category	ß	Std. error	*P*-value	OR (95% CI)
[QOL and wellbeing = 1]		−2.963	1.129	0.009	[−5.176, −0.750]
[QOL and wellbeing = 2]		−2.411	1.122	0.032	[−4.611, −0.211]
Disposable income		0.360	0.118	0.002***	[0.129, 0.592]
Education funds		0.499	0.141	0.000***	[0.223, 0.775]
Discrimination		−0.392	0.168	0.020**	[−0.721, −0.062]
Freedom of expression		−0.475	0.227	0.037**	[−0.920, −0.029]
Gender	Male	0.173	0.352	0.623	[−0.517, 0.863]
	Female	0^a^			
Age-group	30	−0.335	0.347	0.334	[−1.015, 0.345]
	30+ RC	0^a^			
Occupation	Not employed	−0.882	0.440	0.045**	[−1.745, −0.019]
	Employed RC	0^a^			
Education	Not attended any	19.348	0.000	0.000***	[19.348, 19.348]
	Attended RC	0^a^			
Household	Not head of the family	0.813	0.405	0.045**	[0.020, 1.607]
	Head of the family	0^a^			
Income	MK5,000	1.921	0.515	0.000***	[0.911, 2.932]
	MK5,000+ RC	0^a^			
Observation number	402				
PseudoR2 (nagelkerke)	0.363				
Log likelihood	297.66			0.000***	
Test of parallel lines					
−2 Log Likelihood	293.363				
Chi-Square	4.297				
DF	10				
*P*-value	0.933				

*Source: Authors 2021 computed using SPSS version 25.*

***, **, and * means 1, 5, and 10% levels of significant, respectively.

*^a^RC, Reference category.*

The results of −2 Log L (−2 Log Likelihood) for model goodness of fit suggests that the intercept with covariates model was reasonable compared to the intercept-only model. Intercept with covariates model preferred one (best model). Moreover, the overall model fits and evaluates the contribution of each effect to the model. The results of the Likelihood ratio test and Pseudo R^2^ (Nagelkerke) suggest that the model was well fitted to the data.

## Discussion

The present study aimed to find the impacts of the Sharing economy on the individual and community QOL and wellbeing by looking at their associated influencing factors using VSLAs as the model of sharing economy in the least developing countries; a case of Mzuzu city in northern Malawi, southern Africa. The study was conducted in a period usually where VSLAs members share their contributions and start new circles (November–January 2021) in Malawi, which has added value to the insight of our findings as per our study design.

### Overall Findings on Social Demographic Characteristics

Within the scope of social-demographic characteristics, our findings revealed that there is an indication of the progress being made in terms of men in participation of VSLAs or savings groups in Malawi. It has been known that women in many developing economies tend to dominate these sorts of programs wherever they are implemented (Coleman, 1999; Thophilus and Paul, 2019). The rise of male participation could be explained by the fact that the importance of these savings groups has been highly recognized and promoted to be formal organizations by authorities in many economies, including the Malawi government, which could also be a motivating factor. For instance, the programs and strategies that the government of Malawi introduced such as the Financial Inclusion 2016–2020, Malawi National Social Support Programme II- 2018–2023, and Savings and Loan Groups Best Practice Guidelines (SLG BPGs) 2016–2017 have facilitated the creation of VSLA’s by both genders to become a nationwide issue of interest (Reserve Bank of Malawi, 2016; Banda, 2021). These programs have not only helped to positively change attitudes regarding gender and such organizations but also have enhanced the operations of the VSLAs in Malawi and thereby have improved the effectiveness and efficiency of the VSLAs, which at the end has also positively affected the wellbeing of the individual and the community at large as the majority are encouraged and motivated to join these groupings.

Furthermore, there is evidence that the majority of the people had attained a certain level of education, which is encouraging development in this twenty-first century especially in Sub-Saharan African countries like Malawi where education and other sectors of development are still lagging behind (Tambo et al., 2016; Bodomo, 2019). In a global village world, education plays a major role especially when it comes to the sharing economy, as the majority of the transactions are online, and there is a need for one to have a basic level of education in operation. This education is believed not only to help the members within a community to transact but also to live a better life, evidenced by some studies that attest to the fact that those with a good level of education tend to have a better life (Campos et al., 2014; Purba et al., 2021). The findings of this study have also revealed that the majority of the members were relatively mature in age, falling above 30 years of age. It is thus not a surprise that these respondents were also often the head of the family which gave them the responsibility to take care of their offspring. Despite them attaining a certain level of education, the findings show that these members were not employed in formal settings, thus, agreeing to the fact that unemployment rates are still being a challenge in many developing countries including Malawi (Dixit et al., 2011; Garrity and Martin, 2018). However, on the same note, these findings bring and highlight a very important point based on sharing economy: VSLAs allow people to share and enjoy the services within a community despite not having a formal working relationship with one another. This is a way in which a community that has both unemployed and employed as well as educated and uneducated can achieve a degree of equity (New York Times Editorial, 1973; Wong et al., 2020).

### The Role of Village Savings and Loans Associations in Promoting Community Wellbeing

For a decade, VSLAs as a model of the sharing economy has proven to be a tool that has positively impacted the wellbeing of their members and the community at large. Based on the analysis of our study, we have found that there are some factors that are associated with the operations and performance of the VSLAs that have direct and indirect impacts on the wellbeing of the members and community at large.

To start with, our findings based on the regression analysis model have revealed that education is a strong predictor of quality of life and community wellbeing as it was found to be statistically significant (*p* = 0.000). According to literature, the importance of education cannot be underrated within a social setting given its capacity to enhance a skillful and flourishing society (McKibbin, 2017). These findings agree with several other studies that also have revealed that education is one of the factors that significantly contribute to a person’s better quality of life and wellbeing (Campbell et al., 1976; Edgerton et al., 2012). Thus, achievement of better education means having a wider knowledge of public health as well as the potential of having high income thereby contributing to the overall QOL and wellbeing (Campbell et al., 1976). In these current times of the digital world, education facilitates the initiation and completeness of transactions each and every day, meaning that those who have attained a certain level of education, such as those that have attended high school or secondary school, are able to communicate sometimes even without the need of any middle man depending on the type of the business transaction they want to perform. For instance, the majority of sharing economy models like DiDi in China, Airbnb in most of America, Uber, etc. (Sun and Ertz, 2021) all require a certain level of education or knowledge to operate their applications successfully (Akande et al., 2020; Chi et al., 2020). However, our study has differed from another study conducted in Nepal in which they found that education only was not a significant predictor of QOL and wellbeing (Acharya Samadarshi et al., 2021). The discrepancies could be a result of research design differences. In continuation, we have found that there is a positive correlation or association between the availability of disposable funds and education’s members agreed that VSLAs led to an increase in disposable funds which in turn is being used to pay for their education expenses, something which is paramount for human development and individual QOL and wellbeing as per our discussion. Another explanation for this fact is that there is a high probability that most of the educated members of VSLAs had a better QOL and wellbeing due to their exposure or knowledge of public health as well as various means of income-generating opportunities (Campos et al., 2014; Purba et al., 2021). Our findings are also consistent with a study on education and quality of life which found that higher tertiary educational achievement leads to occupations likely to provide the opportunity for continued refinement of the cognitive and interpersonal skills which is also believed to promote community wellbeing (Edgerton et al., 2012). In this context, occupations that require one to have higher educational qualifications tend to provide such people with relatively high earnings which, in turn, enable them to access a wider range of material and non-material resources and opportunities (Davis, 2006). Consequently, higher earnings afford an opportunity for individuals to live a safer and more-resourced lifestyle (Edgerton et al., 2012), which will at the end lead to living a better QOL and wellbeing of an individual that even in the end will lead to a satisfactory community as each and every individual within it are enjoying a better life.

In continuation, our findings of this research also indicate that VSLAs have enhanced the availability of income to their individual members and that said income (Income levels, and disposable income) is a strong predictor influencing individuals’ quality of life and community wellbeing. Firstly, on the income levels, our study demonstrates that income level is one of the strongest associated factors that is associated with QOL and wellbeing based on the two-way ANOVA test, which means that those that earn high have the probability of living a better life as compared to those that are at a low level of income. These results are in agreement with recent studies that were conducted in Vietnam which found that income was highly associated with wellbeing and QOL people during the COVID-19 pandemic (Tran et al., 2020). Income plays one of the vital roles within the life of an individual as it determines the kind of life a person may live, for example, its evidenced in the literature that those who earn higher levels of income have more options on spending in health, good and diverse food types, education, etc. which can also be linked to their wellbeing (Espenshade et al., 1983; Statistics Canada, 2021). An individual living this kind of life will be more productive in his community by either engaging themselves in the activities that enhance the community because of their good health status, satisfactory and happy life. Much as income plays a vital role in a world of sharing economy, it should also be stated that it’s not always through income that someone may share or enjoy the goods or services in the sharing economy. For example, a barber may exchange service with a bicycle operator or rider but in these instances of VSLAs it makes sense for income to be the influencing factor as they are found on share contributions which come in a form of income (Ngegba et al., 2016; Hinson et al., 2017; Agarwal and Steinmetz, 2019). Despite this, a fierce debate exists in some literature regarding whether or not income alone does contribute to QOL and wellbeing (Livingston and Fink, 2003).

On the same note, drawing evidence from the survey, most of the respondents reported that VSLAs have led to an increased amount of disposable income among the members resulting in life satisfaction and happiness to some extent. Some studies have found strong evidence regarding the correlation between disposable income and people’s quality of life and community wellbeing. VSLAs members across the wards felt highly satisfied with the readily available disposable income necessary for emergency support. In line with our findings, a study conducted in Britain using the British Household Panel Survey data (BHPS) found that income had a positive as well as a significant influence on the life satisfaction of individuals (Brown et al., 2004). Scholars argue that in a lifetime, everyone aims at having a disposable income that would be readily available to help in times of need like a health emergency, education, food, etc., which in turn, influences the people to live a better QOL and wellbeing (Campbell et al., 1976). Thus, we argue that readily available disposable income obtained from VSLAs implies that members had access to more finances for basic necessities, thereby reducing household poverty as well as other socioeconomic burdens which also enhanced the overall community wellbeing of Mzuzu City and the country of Malawi at large. Our argument can also be supported with some literature. Another study conducted in northern Malawi found that the introduction of VSLAs led to a rise in people’s living standards and welfare by improving food security and increased household income, which indicated a better QOL and wellbeing among such households and the community (Ksoll et al., 2016).

Furthermore, the findings of our study have revealed that VSLAs have impacted the community QOL and wellbeing through freedom of expression as it was found to be statistically significant when tested in the model using ordinal logistic regression. However, our findings have highlighted a negative impact which has a disagreement with other previous studies which found that the greater the degree of freedom a person has, the happier a person becomes (Haller and Hadler, 2004) and thus may lead to the better QOL and wellbeing of the person. A recent study published in the journal of *Applied Research in Quality of Life* also found a strong link/relationship between freedom and happiness (Abdur Rahman and Veenhoven, 2018). Therefore, we cannot rule out the fact that VSLAs provide a platform for freedom of expression within a community for its members which has an impact on the community as the people are able to express their feelings. The discrepancies of the findings could be explained by the fact that the majority of the respondents in our study especially in developing countries, would have lacked adequate knowledge, understanding, and interest in gender equity and equality issues like freedom of expression, discrimination, women empowerment, etc. In relation to this, our findings also laid the fact that discrimination was a significant predictor of QOL and wellbeing. However, it negatively influenced the QOL and wellbeing among members of the society as most of the respondents were highly satisfied with the coming of VSLAs in Mzuzu city. Reduced discrimination increases one’s potential to fully participate in various activities as they feel a sense of belonging and recognition in a social network or society as a whole (Aggarwal et al., 2015; Coley et al., 2017). VSLAs as sharing economy models are built on the concepts of social trust and social capital. Thus, it’s reasonable for the VSLAs to operate efficiently to avoid discrimination or any related issue that could jeopardize members’ relationships and trust. Our findings are consistent with a study conducted in the United States which found a positive association between poor health-related quality of life, wellbeing, and perceived discrimination (Coley et al., 2017). There is huge evidence that some members of VSLAs do experience discrimination in their communities for various reasons and their lives change for the better through VSLAs meetings. Given that humans are social beings, interactions with families, relatives, and friends are crucial for mental health such that through socialization, one can deal with stress that comes due to loneliness, hence contributing to their overall QOL and wellbeing (Campbell, 1976). When an individual is satisfied that is being valued within a community, it gives the person a motivation factor to work hard in the promotion and overall engagement in the community services. Thus, eradicating discrimination which has come about through VSLAs not only the social status affirmation of the members but also promotes community development and wellbeing.

### General Contribution to Sharing Economy

Our study has emphasized the importance, and the role played by the trust as an element in sharing economy model for them as a system to work. This emphasis enlightens us that trust not only facilitates the sharing economy as pointed and emphasized in many works of literature available (Maganga, 2020; Köbis et al., 2021). However, trust goes beyond that edge by acting as a core domain at every stage of the sharing economy, starting from the early stages of transactions initiation where the consumer or the village bank members decides to join a certain group, throughout its circles and the enjoyments of the service/a good, to the end of the returning the service or a good, in this case, if it’s in regards to VSLAs it may encompass to the returns of shares and sharing of the profits at the end of the circle of the group within the community.

## Limitations

The study was conducted during the agonizing time of COVID-19 and there were a lot of social-economic challenges faced. Precisely, most participants complained about little income to purchase data for the internet as the cost of it is prohibitively expensive in developing countries like Malawi. However, we overcame this by reminding the respondents that they could fill in the questionnaire when they had the data as long as they did so within the data collection timeframe. Secondly, the respondents were not motivated as many people expect a token of appreciation when involved in surveys. Despite this, we tried to minimize this limitation by informing the participants that they could win a rotary on a raffle draw and that the groups that had many respondents would be acknowledged and rewarded at the end of the study.

Lastly, this study used a cross-sectional design that is also limited in identifying associations and casual relationships.

## Conclusion

This study has, for the first time, examined the impacts of the Sharing economy on the individual and community Quality of Life (QOL) and community wellbeing by looking at their associated influencing factors using VSLAs as the model of sharing economy in Mzuzu city, northern Malawi. We conducted our study from November 2020 to January 2021 using a cross-sectional survey design involving a total of 402 participants recruited through snowball and respondent-driven sampling techniques.

Our study found strong evidence regarding education status, thus, those that are said to attain a better education (starting from college-level above) are said to have a better QOL and wellbeing as compared to the other due to the fact that education enhances income opportunity, knowledge to public health-related issues, etc. Disposable income, thus, those that have enough disposable income were able to live a better life as they were able to manage good education, pay for their medical bills, manage to acquire assets like wireless, Tv, Iron sheet, houses, etc. which in turn improved their QOL and wellbeing. Freedom of expression and discrimination thus, VSLAs as social groups gives a chance to the members for an association, giving them a say and refraining from discrimination helped them to have a sense of belonging and a voice which leads to a better QOL and wellbeing of the members and the community at general as significant factors that influence the quality of life among VSLAs members. However, we found an absence of evidence as to whether gender and age of the respondents have any influence on QOL, this is so as a scholar has pointed out that the fact of just being a male or female does not have an impact on someone’s QOL and wellbeing (Lynch et al., 1998; Fodor et al., 2011; Joshanloo and Jovanović, 2018; Acharya Samadarshi et al., 2021), but there are a lot of the attributes that are involved like education, income, etc. Therefore, it can be concluded that the findings of our study also show that VSLAs play a sterling role in promoting the quality of life and wellbeing by lifting people out of poverty through increased income, education, and social inclusion.

Based on the findings, there is a need to replicate the study that will consolidate on using a comprehensive approach that will consolidate our findings for effective policy implications to improve people’s overall living standards. Furthermore, we recommend Micro financial institutions like banks in all developing countries like Malawi to educate the VSLAs members about the importance of opening bank accounts and introduce strategic plans to accommodate these VSLAs members like opening outlets in rural areas and introducing mobile banking.

Lastly, we further recommend the authorities to reintroduce and improve those policies like that of National Strategy for Financial Inclusion 2016–2020, Malawi National Social Support Programme II- 2018–2023, and Savings and Loan Groups Best Practice Guidelines (SLG BPGs) 2016–2017 which were aimed at increasing the participation of VSLAs members in Malawi by providing basic business training, expanding VSLAs coverage through social cash transfers and facilitating their registration, etc., and establishment of the Malawi national VSLAs implementation, respectively.

## Data Availability Statement

The raw data supporting the conclusions of this article will be made available by the authors, without undue reservation.

## Ethics Statement

The studies involving human participants were reviewed and approved by the School of Economics and Management of Yangtze University (Approval number REF/YU/2020/08) and Mzuzu City Council (Approval letter reference number MCC/dated on July 20, 2020). Written informed consent to participate in this study was provided by the participants.

## Author Contributions

X-LW and GM contributed to the conceptualization, validation, visualization, project administration, and methodology. GM, AA, and WS contributed to the software, data curation, and formal analysis. MD, GD, and YS contributed to the investigation, writing, reviewing, and editing. X-LW and MR contributed to the resources and supervision. GM contributed to the writing—original draft preparation. All contributing authors have read and agreed to the published version of the manuscript.

## Conflict of Interest

The authors declare that the research was conducted in the absence of any commercial or financial relationships that could be construed as a potential conflict of interest.

## Publisher’s Note

All claims expressed in this article are solely those of the authors and do not necessarily represent those of their affiliated organizations, or those of the publisher, the editors and the reviewers. Any product that may be evaluated in this article, or claim that may be made by its manufacturer, is not guaranteed or endorsed by the publisher.
